# Mental Health and Access to Information in Times of COVID-19: The Role of Social Work

**DOI:** 10.3390/ijerph19084483

**Published:** 2022-04-08

**Authors:** Ana C. Romea, Diana Valero, Carmen Elboj, Patricia Melgar

**Affiliations:** 1 Faculty of Communication and Social Sciences, San Jorge University, Villanueva de Gállego, 50830 Zaragoza, Spain; acromea@usj.es; 2 Department of Psychology and Sociology, University of Zaragoza, 50009 Zaragoza, Spain; celboj@unizar.es; 3 Department of Pedagogy, University of Girona, 17004 Girona, Spain; patricia.melgar@udg.edu

**Keywords:** social work, mental health, socially vulnerable groups, COVID-19, pandemic, health inequalities, social intervention, ICT

## Abstract

The confinements and limited social interactions that have derived from COVID-19 have aggravated the situation of people with previous pathologies. As a result, access to health and its rehabilitation or support resources has been limited and redirected toward online care. People with mental health problems have considerably suffered during the pandemic because, for many of them, accessing different information resources through telematic means proved to be a great difficulty in their everyday lives. This exploratory research work aims to establish which elements have successfully facilitated access to online information for people with mental health problems. This is especially urgent in times of COVID-19 when misinformation has significantly harmed this group. To do so, we followed the communicative methodology and held interviews with two professionals and nine users who participated in the case study. The main results show that, in developing digital communicative competencies in accessing information with this group, individualization of care to overcome barriers, the link with social workers as references of truthful information, and the importance of emotional work and interactions are critical elements.

## 1. Introduction

The COVID-19 pandemic is one of the worst in the world in memory. It has had obvious consequences on the entire population’s physical health but, at the same time, is also causing long-term consequences for mental health [1,2,3]. For those who already had a mental illness, the exceptional measures taken disproportionately especially affect the most vulnerable groups [4,5].

Some of the preventive measures imposed to prevent the spread of this disease, such as social distancing, recommendation or prohibition of large gatherings of people, and above all, home confinements, are those that most strongly impact people’s mental health [3]. Research indicates that these health crisis situations can lead to isolation for the entire population, decrease physical and mental activity, increase recursive thoughts, aggravate already existing mental illnesses, and augment stress and anxiety disorder that therefore bring about a growing demand for mental health services [6,7,8,9].

However, one resilient aspect is how information and communication technologies (ICT) have become allies during this pandemic by favoring social contact, facilitating shopping without leaving home, generating opportunities for leisure and entertainment, allowing telework and tele-education, and transmitting information [10,11]. Between 2019 and 2020, the time that people spent every day using the Internet on any device increased by 4%, and the use of social networks rose by 13.2% [12]. ICT have also been applied to numerous fields, including medicine and mental health, and have proven to be a helpful resource that is demanded by professionals and patients alike [13] However, not all the population has benefited equally from ICT. One of the cases of unique vulnerability is mental health patients. They have faced barriers in accessing ICT, which can lead to double social exclusion due to their health condition and their health difficulties in such access.

This is an exploratory study in which our aim is to identify the difficulties of people with mental health problems when handling information during confinement and, especially, the elements that have allowed them to overcome these barriers.

## 2. Background

Many studies show that ICT can facilitate people with mental health problems [14]. For example, ICT are helpful for improving these patients’ cognitive capacity [15,16], they can be applied to the educational field [17], and they have positive effects on facilitating these patients’ social and leisure activity [18,19]. Today, ICT have become an indispensable element in people’s leisure time because their use may already constitute a hobby, and they allow people to connect to hobbies of all kinds [20]. Furthermore, connecting people with mental illness to ICT is positive [21], and digital social work can potentially implement innovative mental health interventions [22,23,24].

However, some barriers prevent this group from accessing the use and practice of ICT on equal terms: associated stereotypes [25], the course of their disease itself [26], lack of training in digital skills [27], costs of digital technologies, and no showing interest in using them [28]. It is essential to point out that some barriers that hinder ICT access for people with mental illness can be overcome with appropriate support and adaptations [29].

During this pandemic, the vital role that ICT played in accessing information for the entire population was evidenced [3], including, of course, in the mental health field. Many studies are investigating the use, benefit, and impact of ICT during the pandemic. For example, messaging apps have been crucial for helping people deal with mental health issues, especially those related to isolation and stress. With them, they have been able to receive information about COVID-19. They are also a good option because their use does not require high digital training, and thus, vulnerable groups can employ them [30,31]. Furthermore, official and social organizations often resort to this platform to send messages with reliable information [32,33].

Social networks have also played a significant role for this group during the pandemic, but their use in mental health may imply certain controversy. Studies suggest two positive aspects of social networks for users with mental health problems: some people with mental health problems have utilized social networks to communicate and make their experiences visible, and handling social networks can be an excellent way to face social isolation [34]. However, the idealized image that is often projected on social networks can negatively affect those who compare themselves to others, which increases stress and anxiety [35]. Finally, hoaxes of all kinds can be quickly spread via social media [36,37].

Exposure to the mass media during COVID-19 can increase symptoms of depression, anxiety, and hypochondria, especially in those groups that were already vulnerable to start with [38], including, of course, users of digital media.

Many people have resorted to the Internet and social networks to seek information and to feel specific security. Unfortunately, a considerable increase in hoaxes and deliberate misinformation has made people’s mental health worse [39,40], which has evidently worsened the mental health of people with a previous mental pathology [41,42].

According to data, Internet users voice many concerns about fake news: 56% of adults aged over 18 years are uneasy about distinguishing between the real and false news they receive over the Internet, and this percentage rises to 65.1% in Spain [12]. Therefore, administrators must make commitments to provide reliable information to prevent this misinformation, especially for this vulnerable group [43]. For this purpose, health organizations and the third sector of social action have been crucial in preparing documentation and distributing it to its users [44,45].

Basically, insofar as ICT have been used as a means to report the pandemic, a direct and intense relation between inequalities in accessing digital technologies and COVID-19 exposure has emerged [46]. This aspect is especially significant for people with mental health problems because, in some cases, the medication they take for their illness affects their cognitive abilities, which makes people more vulnerable to hoaxes [47]. This is why it is essential to establish successful strategies when developing programs and policies that allow the population in general, and people with mental health problems in particular, to access information to develop their digital and communicative competence.

## 3. Case Study

The case study was carried out in a mental health center in a city of northeast Spain. All the users of this center have some mental health problems. If they go to the center, it is because they are in a situation of psychopathological stability, which allows them to be incorporated into a social inclusion process to acquire and recover the necessary skills and abilities to improve their functioning in the leisure area.

The activities offered by this center vary and aim to meet the leisure needs adapted to this group (sports, artistic, cognitive improvement, dedicated to the ability and managing of communication technologies, promoting autonomy). The methodology that characterizes this center’s activities is motivating and participatory, in which volunteers, social workers, and users collaborate throughout a design, development, and evaluation process.

When the Spanish Government decreed confinement due to COVID-19, this social club had to close its physical space. However, some of its 90 users have no other mental health resource, and it was essential to continue its activity electronically by reinventing intervention in leisure using ICT as a fundamental basis.

This center was selected because although, in the city chosen for the study there are more leisure centers for people with some type of disability, this center works exclusively in the field of mental health, with no users who have another type of disability, which allowed us to delve into the limitations that affect this group. Of all the activities that are carried out in the center, the photography activity was chosen to be analyzed since it is the one that entailed a greater integration of ICT.

## 4. Materials and Methods

The communicative methodology was followed for this exploratory case study. This methodology allows shared knowledge to be created between research and citizens. The scientific knowledge provided by research and participants’ lived experience is combined to generate shared knowledge that has a stronger social impact. This is a transformation process, which makes it especially valuable when working with vulnerable groups because it favors empowerment processes [48,49,50]. Following this methodology, the transformative dimension, which allows barriers to be overcome, and the exclusion dimension, which refers to the barriers that prevent people from enjoying certain social spheres, were employed as dimensions of analysis in this context. They were those that would make it difficult for the social club participants to use ICT and access information. At the same time, the transformative dimensions include the elements that facilitate overcoming these barriers [51].

Specifically, 11 semi-structured interviews were held with users and professionals. Given the health situation, interviews were conducted by telephone or by different ICT adapted to users’ technology availability and their degree of knowledge. Finally, the chosen means were WhatsApp and Skype phone calls and video calls.

The purpose of the interviews was to know which elements facilitated the development of users’ digital and communicative competence. These two skills are essential for accessing information. Digital competence “consists of having the skills to search, obtain, process, and communicate information, and to transform it into knowledge. It incorporates different skills, ranging from access to information to its transmission in different media once it has been processed, including the use of information and communication technologies as an essential element for information, learning, and communication” [52] (p. 38). This competence includes searching, selecting, and analyzing information, including digital media management knowledge.

Communicative competence, in addition to knowledge of a language and the ability to transmit and understand it, “implies that the mere consideration of the function of language as a possibility of naming must be transcended, also taking into account its relational, communicative and expressive aspect: lament, rejoice, attack, defend oneself, persuade, express intentions and needs, etc., within a specific social and cultural context” [53] (p. 9).

These two competencies are essential for developing social and civic competence, which apart from being one of the social club’s purposes, is essential for all people, including those with mental health problems.

### 4.1. Sample

The study consisted of interviewing nine participants and two social club social workers. Information was collected from September–October 2020, which was when activity ended, and healthcare restrictions allowed the leisure center to reopen. Although there were twelve participants in the photography experience, only nine directly participated in the study because their health processes do not always allow them to remember the necessary information or to effectively communicate it. Table 1 provides a summary table with the interviewees’ main characteristics.

### 4.2. Data Analysis

For the analysis, we used the communicative methodology, and following this, Table 2 presents the pattern of coding performed for this study. Specifically, data were coded as two large categories: digital competence and communicative competence.

For both these two large categories, each user’s level of competence before the activities performed during the confinement period and the level acquired after the intervention made during confinement were analyzed. This gave subcategories “pre-intervention” and “post-intervention”.

Following the characteristics of the communicative methodology, these categories were analyzed by considering the exclusionary and transformative dimensions (those that serve as the basis for proposing the most successful strategies).

### 4.3. Ethics in Research

The research methodology was validated by the Ethics Committee of the San Jorge University (Spain) following the principles of confidentiality and guarantees of all the participants, and the case-study center’s consent was obtained to conduct this research. Therefore, all the participants had the legal capacity to act and were able to sign informed consent to participate in the research. As an additional measure, during interviews, they were accompanied by a social worker from the social club to facilitate communication, to understand questions, and to favor a climate of trust.

A code for each participant was generated to guarantee the anonymity of responses. For example, social workers were identified as SW1 and SW2, while users were named P1, P2, etc., up to P9.

## 5. Results

During the pandemic and confinements, the ways to access information also varied in this population. Although access to information used to be via traditional means, which implied gaps or loss of information, access through social networks and other means increased during confinements, which means that those who already accessed information through social networks and other applications could suffer “infoxication”.

“Before the pandemic, it was normal for most of them to do so through traditional media, such as radio or television. But now, of course, not all information arrives through these means, but also through social networks. Nevertheless, what happens to those who are informed through networks is the exact opposite; that is, they now have too much information.” (SW1)

The interviews allowed us to identify three elements to fight against this situation and can guide future programs or policies, namely individualization of care to overcome barriers, link with social workers as information references, and importance of emotional work and interactions.

### 5.1. Overcoming Barriers through Individualization of Care

The field of mental health is extremely heterogeneous, as everyone has, for example, different recovery processes and social and economic factors. Hence, so many barriers have been identified because they also respond to this same heterogeneity.

One of the main barriers that users face with ICT is their starting digital knowledge because many of them have no previous experience or have not sufficiently come into contact with information technologies.

“I handled myself normally, but not too much.” (P6)

“They changed my mobile, and now I have one that has WhatsApp, but it didn’t have it before.” (P3)

This prior knowledge that users had regarding ICT tools refers to the social club, which offers various courses for which users can register. This digital literacy work started before the pandemic. During these courses, both digital and communicative competences focused fundamentally on leisure and free time. However, social workers identified an unlevel use of courses:

“At times, and from one course to the next, they had forgotten what they had learned. Sometimes this is due to illness, but at other times it is because they don’t have the means at home, so they can’t practice. Well, of course, some who don’t want to practice later … So then we have different levels in courses, which is inevitable; we do what we can to adapt to what we face.” (SW1)

“Many [clients] lack practice because they have not been practicing the many courses we have done before.” (SW2)

This previous technology use level is linked with several factors that complicate access to digital technologies: age, socio-economic issues, or users’ rehabilitation process. Specifically, age is a barrier that influences the ability to handle ICT. For example, users P2, P3, and P7 had no difficulty, which the social workers agreed on. However, older users are more likely to come across difficulties in ICT use.

“No, I have always handled technology well, I have no problem.” (P7)

Another barrier that coincided with many users was an economical one. Many of them do not work or have never done paid work. Most receive some type of income or benefit. Some families cannot afford to buy devices like a smartphone or computer or pay fees to talk on a mobile phone or use mobile data. This sometimes prevents these users from having them at their fingertips.

“As I don’t have a mobile, I took the photos with a normal camera. I talked from a landline, and they told me what to do. And then I couldn’t send photos for the exhibition. When we were able to leave home, I took the photos to the social club and we saw them there.” (P5)

Finally, the rehabilitation process or the course for improving mental health also determined the ability to manage ICT. Perhaps not directly but instead transversally, it is a barrier accompanied by other factors, such as the health resources they can access, the support they receive from their families, and the other listed characteristics. It is precisely in these cases when mental health interventions from leisure and free time can substantially contribute to the process of individuals:

“For some, this social club is their only resource … because they have already left other itineraries, or there are not many more offers because the user doesn’t want to go to another place, or for families … During confinement, the mental health of some got worse. Not all, but some. Yet for others, this time helped them to clear their minds, and so on. However, for some, talking about those days is impossible because they no longer remember … and for others it would not be appropriate to do so right now.” (SW1)

Such interventions from ICT in these users’ leisure field have often been fundamental to favor their recovery process by providing escape routes that mitigate the effects of confinement.

“I handled it well. Yes, I handled it well, but in the end, I ended up being admitted [to a mental health facility]. Not too bad, but I ended up with depression, and in the end, I ended up being admitted. And I was playing video games, listening to music, watching a movie … a bit of everything, but also … nothing else … Something from the social club too.” (P7)

The critical element that was able to overcome and transform all these barriers was the social workers’ individualization of care for users. To begin the practical intervention before confinement, the professionals had to start with each user’s resources. To do so, they rang each user to consult the means available to them. They made a list, which allowed them to send information to users according to their selected medium with these data. In some cases, they had to hold short tutorials or prepare manuals to help users to update or download applications, such as WhatsApp or Skype, and other messaging applications.

“One of the first things we did was to make a list of the devices that users had and the applications that each one knew how to use. We even made some simple manuals for someone to learn how to download WhatsApp or to update it. But, unfortunately, we only had landlines for some of them because they had to choose between paying for data and other priorities. With this, we were able to adapt, and information and activities reached everyone.” (SW2)

In other words, adapting the interventions to each user’s characteristics guaranteed everyone’s ability to access the activities, information, and ICT. This was why it resulted in their recovery and empowerment process.

“I just never had to use it before. So let’s see, the mobile camera, yes, because I had signed up for an activity that we did before the first confinement. But then they told me how to watch the news on my cell phone also in passing.” (P9)

“I don’t have WhatsApp or data. They told me the letters for the photography exhibition, and I took photos of the objects or things that began with the letter … They had to tell me over the phone. We did everything by phone.” (P1)

### 5.2. Link with Social Workers as References of Truthful Information

One of the critical elements of this intervention was that from the first confinement, the social workers provided users with accurate information about the pandemic and its development. This meant that the social workers acted as references of truthful information for users who, despite receiving much information on television or via social networks, had these professionals as references to trust.

“At the beginning we had no idea what was going to happen, thinking that it (confinement) was going to last 2 weeks or so. So information about the pandemic and such was sent. Then as we realized it (confinement) would last longer, we began transforming our intervention. At any rate, we never stopped sending information about the pandemic.” (SW1)

As one of the users pointed out, the professionals referred them to all the necessary information about COVID.

“They sent us information, well, about washing our hands, staying at home, coughing in our elbow … and if it was COVID or it was not COVID. Let’s see, what else? Well, everything about COVID.” (P2)

It is highlighted that not only were users contacted to send information about COVID, but information about the pandemic was also included in their activities. In this way, information reached users more naturally and with the same impact when indicating the activities to be performed.

“Well, they gave me mandalas to color in; I colored in many mandalas to encourage the elderly in a nursing home. And also about staying at home and about COVID things. Ah! And of course about the letters that we had to write.” (P4)

They also reinforced the fact that users were explained to not trust hoaxes but to only trust reliable sources so as to not mix false information with the truth.

“The hoax thing has not gone unnoticed, no it has not. There were hoaxes, of course, but I do not know if they reached all the users or not. We told them to trust what we sent them.” (SW2)

The information users received involved several functions. In addition to the health function, it also sought to mitigate hoaxes and to improve users’ critical and reflective capacity, which ultimately helped them to reflect on the veracity of the information they received by other means.

“They can fall for hoaxes because many factors come together. That is, myself, my mother, or anyone can fall for hoaxes. We can pay attention to them or ignore them. It is not a matter of them being more likely to fall for hoaxes, but it possibly being different for them because hoaxes can affect their health or recovery processes […]. They were explained what was true or what was not. When we ran courses, sessions, workshops, etc. on technology management, they were told about this issue. For example, not everything that Google tells us might be true.” (SW1)

### 5.3. Importance of Emotional Work and Interactions

During confinement, the general population experienced increased anxiety and also suffered mental health problems. Therefore, it is essential to find a way for users to express their emotions. Some users could not continue with their therapies in the normal manner, and others or their relatives were affected by COVID-19, and abandoning routine for some could pose problems for their mental health. For all these reasons, a decision was made to include the production of texts and user reflections in the activities that they already performed before the pandemic but were more playful in nature, such as photography. To adapt this to remote work, each patient was given one letter. With it, they had to take a photograph of something that started with that letter and accompany it with a short text about what had inspired them. In this way, they worked on their emotions and expressed how they felt while developing their digital and communicative competence. Two of these users described this as follows:

“Well, for example, keys as they inspired me precisely because we were all locked in, to see when quarantine ended in quotation marks and to be able to go out and open all the doors that had been closed to us until then. And that inspired me; let’s see if this happens soon. Then I also had “yummy-yummy” because, apart from the fact that we always celebrate everything with food and, by also being also in lockdown, I think that everyone has made more trips to the kitchen … but my idea was to also use something healthy, but also rich, in the photo, and something sweet too … So that’s why I put a little chocolate in the photo, something that gives us a little joy too. That was in the Ñ. (P8)

Well, I got the J, and I took some pictures of a few dolls that I have on the floor, because dolls are toys and they evoke nostalgia, well I took them. I took a photo of some jewelers and of some tulips that I have in a vase because that makes me feel serenity”. (P1)

The writing of these texts required social and family workers’ accompaniment. They invited users to reflect on the events that they were experiencing so they could even offer the above-mentioned information about the pandemic course through this dialogue and reflective process.

“I got an R. Well, in principle, I was going to take a picture of a clock. But I also thought of a souvenir from Belgium, from the time my sister worked there. But my mother told me that as I had had this idea, and it was good one, I shouldn’t change it. The explanation of the clock issue is because the hours passed by very slowly and I was looking at the clock every 2 or 3 h. Once we were able to go out, even for limited hours, things changed because both my father and I fought a lot about confinement. We handled it very badly.” (P9)

“It does not seem so, but it was a way of channeling the information that these users received externally. If we detected in the texts that someone could have an idea about the coronavirus or wrong confinement, we had room to redirect them.” (SW2)

This specific activity ended with a virtual photograph exhibition. The inauguration was participatory, as users were once again able to find spaces to express themselves and share experiences, in other words, placing users at the center of the intervention and also at the forefront of their recovery process. The complete photography activity intended to cover the need to promote users’ critical and abstract thinking, that is, to improve their communicative competence.

“I saw the photos of my colleagues. I went to the opening and saw the photos. I liked the landscapes, the images were very positive for me, as were the ways to hang photographs … It was a very dynamic exhibition, very pleasant to see and it was short. And then, what I said that day, well, that’s how we express ourselves.” (P4)

“Being able to express ourselves and bring out what we hold inside. It was very long, sometimes sad … but I take that with me. The chance to be able to express ourselves.” (P6)

## 6. Discussion

Mental health patients have been an especially vulnerable population during the COVID-19 pandemic due to isolation measures, while limitations for interactions can negatively influence their care and care processes. With these confinements, care has been generally adapted for its provision through telematics means, and telecare has been vital for mental health patients [37]. Furthermore, telecare reinforces vulnerable groups’ access to ICT, which can help to reduce the impact of COVID and exposure to the risks of this disease [46]. Moreover, it positively affects their mental health [21] and provides educational and leisure opportunities [29]. Hence, contributing to develop patients’ digital competence is essential, and as part of it, access to information plays a preponderant role.

The knowledge that these users generally acquire when handling ICT tools and how to access and communicate information favor this group’s equal access to information by, for example, preventing their vulnerability to hoaxes and by also giving them communication tools. In this way, all patients, especially the most vulnerable ones, acquire guaranteed digital training and the necessary digital skills, which is a matter of justice and social equity [31], as this is the main need detected as the purpose of our research.

If access to truthful information is important for the entire population, it is also very relevant for this group because it has to face major barriers when accessing information linked with the digital competence. This group specifically faces entry barriers, such as financial limitations when accessing devices or the Internet and barriers related to their prior knowledge. Some authors have pointed out that these barriers can be eliminated by employing more straightforward, cheaper, and more flexible technologies because their potential for working on mental health is on the rise [18]. However, in light of our results, accessing these simpler, cheaper, and more flexible technologies requires a further step in program and policy development. Contributing to breaking these barriers, which is the second part of the objective of this research, leads us to the proposed results; specifically, overcoming these obstacles requires individualization of care, which is provided by the professionals who participate in caring for users. We cannot speak of a homogeneous group with the same barriers because their disease process is different. Therefore, it is essential to individualize care when overcoming barriers. At this point, e-social work can be a helpful factor in mental health interventions, as it allows the flexibility and adaptation of interventions with these users, which demonstrates the potential of digital social work to work on mental health issues [24].

We can link this with our second result, which is, in turn, linked with social workers as information references. These professionals’ work mitigated the adverse effects of substantial exposure to mass media and infoxication during the pandemic, which cause depression and anxiety, especially in those with pre-existing mental pathologies [38], and is also effective to avoid COVID-19 hoaxes from spreading. Thus, on the one hand, future programs and projects should consider that in order to deliver relevant information to people with mental illness, professionals with a previous link should be relied on, and attention should be paid to strengthen that link. On the other hand, the importance of this professional figure lies in the fact that the information they transmit is always truthful, and given this scenario, a high risk is posed if these professionals spread hoaxes.

Finally, when fighting the infodemic, emotional work and interactions are essential because expressing feelings helps to mitigate anxiety. Of all the possibilities offered by ICT, we focus on the opportunity to explore ways of expressing emotions, as other authors [17] have already pointed out. One of the issues that professionals and users highlight as being unfavorable is that although ICT have alleviated the effects of isolation during confinement by allowing communication, incorporating more socializing elements would have improved the experience. In this expression of feelings, and given some users’ communication difficulties, the support of both family members and professionals is essential. Similarly, it is vital to facilitate interactions with people and to practice these skills for people with mental health problems.

## 7. Conclusions

Albeit exploratory, this work acts as a starting point to establish which elements can improve digital competence and access to information for people with mental health problems, an area that has barely been explored to date. The results presented focus on the second part of our objective, which is specifically to identify the elements that have made it possible to overcome the difficulties in accessing information and, in this way, contribute to the improvement of their digital skills. The identified elements are also transferable to other populations because the barriers they face are often the same. However, it is necessary to conduct further research because very few articles focus on this group.

In particular, we propose that future research could focus on the training of social workers in the use of ICT and specifically in the recognition of fake news since they are the mediators between information and vulnerable users; moreover, it would be possible to delve into how fake news affects users’ health processes (increased stress, anxiety, etc.) as well as the most appropriate prevention mechanisms. In addition to aspects pointed out by authors such as Miranda [54] regarding the necessary mental health training of social workers, it would be interesting to include digital competence and digital social work issues in their initial training.

Of all the limitations, we highlight diversity in communication with the participants. Given the diversity of mental health problems, interviews were complex due to their level of communicative competence, which made it difficult for them to better express themselves. However, we consider that incorporating their voices was fundamental, as it is a traditionally silent group, and incorporating them into research works implies enrichment despite the difficulties faced. In addition, participation in this case study showed that intervention in social work through ICT, in which the digital and communicative competence is enhanced, can provide people in vulnerable situations with new empowerment tools. Placing them at the forefront of their process of change helps them to avoid suffering a double-exclusion process due to their mental health situation and their restricted access to ICT and information. Finally, given these limitations, we propose a third line of research that would consist of deepening the results through a larger sample that allows us to establish categories within the group itself to avoid its treatment in a homogeneous way and tend towards individualization also from the investigation.

## Figures and Tables

**Table 1:** Interviewees’ main characteristics.

Code	User or Professional	Gender	Age	When Starting at the Social Club
P1	User	Female	45	January 2020
P2	User	Female	33	May 2012
P3	User	Female	29	June 2019
P4	User	Male	53	November 2018
P5	User	Female	53	June 2019
P6	User	Male	49	June 2017
P7	User	Male	36	October 2019
P8	User	Female	64	September 2017
P9	User	Male	49	November 2018
SW1	Professional	Female	37	January 2011
SW2	Professional	Female	30	January 2017

**Table 2:** Coding categories.

	Digital Competence		Communicative Competence	
	Pre-Intervention	Post-Intervention	Pre-Intervention	Post-Intervention
Transformative dimensions	1	3	5	7
Exclusionary dimensions	2	4	6	8

## Data Availability

The datasets generated for this study are available on request to the corresponding author.
